# Wright’s Solution

**Published:** 1914-12

**Authors:** 


### ABSTRACTS

---

**Wright's Solution,** wound dressing causing hyperaemia. Sodium citrate, ½%; sodium chlorid, 3%; water to 100%.

